# Clinical and genomic characteristics of hypervirulent *Klebsiella pneumoniae* isolated from throat culture suggesting an emerging causative agent of pharyngitis

**DOI:** 10.1186/s12879-026-13654-3

**Published:** 2026-05-26

**Authors:** Gregory D. Whitehill, Shekina Gonzalez-Ferrer, Allison T. Tsan, Shangxin Yang

**Affiliations:** 1 Division of Infectious Diseases, David Geffen School of Medicine, University of California, Los Angeles, CA USA; 2 Department of Pathology and Laboratory Medicine, David Geffen School of Medicine, University of California, Los Angeles, CA USA; 3 UCLA Clinical Microbiology Laboratory, 11633 San Vicente Blvd, Los Angeles, CA 90049 USA

**Keywords:** Hypervirulent *Klebsiella pneumoniae*, HvKp, Pharyngitis, Whole genome sequencing

## Abstract

Hypervirulent *Klebsiella pneumoniae* (hvKp) is rarely implicated in non-invasive infections. We report eleven cases of pharyngitis with whole genome sequencing of throat culture isolates identifying hvKp. Most cases (64%) occurred in the summer months of June to September. Cases were geographically diverse and only one patient reported recent travel outside of the continental US. No patients were diabetic or immunosuppressed, though five patients (45%) were prescribed inhaled or intranasal corticosteroids. Clinical presentation was indistinguishable from viral or streptococcal pharyngitis. All isolates were string-test positive. Genetic serotyping and multi-locus sequence typing identified four unique strains: K2–ST3252 (*n* = 4 cases), K1–ST23 (*n* = 3 cases), K2–ST66 (*n* = 3 cases), and K2–ST2039 (*n* = 1). Virulence biomarkers *iucA, iroB, peg-344,* and *rmpA* were present in all isolates, with *rmpA2* only in ST23 isolates. Four hypervirulence-associated plasmids were identified, with ST23 isolates additionally harboring antimicrobial resistance associated plasmids. All isolates carried fluoroquinolone-resistance genes *oqxA* and *oqxB,* and ST23 isolates expressing beta-lactamase (*bla*_*SHV-187-like*_) and fosfomycin-resistance genes (*fosA6*). Despite the presence of genetic resistance markers, all isolates were phenotypically susceptible to common antibiotics. Single nucleotide polymorphism (SNP) analysis revealed high genetic similarity (<2 SNPs) among three ST3252 isolates from the same family, while other isolates showed greater diversity. All patients received oral antibiotics with resolution of symptoms, and post-treatment throat culture showed eradication of hvKp in three of four patients tested. Invasive disease did not develop in any patients. Our study proposes a causative association between hvKp and symptomatic pharyngitis as it circulates endemically in the community.

**Supplementary Information:**

The online version contains supplementary material available at 10.1186/s12879-026-13654-3.

## Introduction

Hypervirulent *Klebsiella pneumoniae* (hvKp) is a pathotype of Kp known to cause severe community acquired infections such as liver abscess and endophthalmitis in otherwise healthy individuals [1]. Even when isolates are susceptible to standard antimicrobials, these infections are morbid and difficult to treat [2]. Clinical reports of invasive hvKp infections abound existing peer-reviewed literature, leading to a putative designation of ‘typical’ hvKp disease as bloodstream infection complicated by deep tissue abscesses and metastatic spread [1, 3]. Non-invasive hvKp disease is much less frequently described but may be more common than previously realized due to 1) the cost- and labor-intensive methods necessary to differentiate hvKp from classical Kp (cKp) strains and 2) a bias in preferentially applying these techniques to Kp isolates associated with ‘typical’ hvKp presentations. Whole genome sequencing (WGS) to identify plasmid-borne genetic determinants of virulence which enhance capsule production (e.g. *rmpA*, *rmpA2*), encode siderophores (e.g. *iroB*, *iucA*), or perform other functions (e.g. *peg-344*) is necessary to accurately identify hypervirulent strains [4]. Case series and epidemiologic studies chronicling hvKp disease in the United States have accordingly selected Kp isolates cultured from sterile sites and primarily from hospitalized patients when screening for virulence [5–8]. However, Kp is a ubiquitous and versatile pathogen which causes a diverse range of diseases, including non-invasive disease in non-sterile tissue sites such as pharyngitis [9, 10]. We propose that hvKp may also cause noninvasive infections such as pharyngitis and that studying these syndromes is important to understand the epidemiology of this emerging public health threat. HvKp pharyngitis has been described in a two prior case reports, one from Germany and one from our institution [11, 12]. Here, we describe the clinical, epidemiologic, and microbiologic features of eleven cases of pharyngitis in Southern California where hvKp was isolated from throat culture and interpreted as the causative pathogen.

## Materials and methods

### Case identification and clinical characteristics

HvKp pharyngitis cases were opportunistically identified in our institution from January 2024 to March 2025. Criteria for a case of hvKp pharyngitis were 1) chief complaint of sore throat, 2) isolation of hvKp from throat culture. Throat culture specimens collected in ESwab (Becton Dickinson, Franklin Lakes, NJ) by practitioners in the UCLA Health System were submitted to the UCLA Clinical Microbiology Laboratory for standard-of-care testing. Specimens were transported by couriers within 24 h of collection and processed upon arrival. Samples were inoculated onto routine media consisting of blood, chocolate, MacConkey and streptococcus selective agar plates. String test screening was performed on all Kp isolates, and string-test positive isolates were further analyzed by an in-house WGS test [7]. Practitioners were notified if hvKp was isolated from cultures. Treatment was left to the discretion of each patient’s provider. Clinical characteristics were retrospectively collated by independent chart review. This study was reviewed by the UCLA Human Research Protection Program and received an Institutional Review Board exemption.

### Bacterial identification, string test, and antimicrobial susceptibility testing

Bacterial culture and species-level identification by MALDI-TOF (VITEK MS, BioMérieux, NC, USA) was performed as previously described [7]. “String-test positive” isolates were determined by formation of a >5 mm viscous string when stretching a fresh bacterial colony with an inoculation loop as previously described [7]. Antibiotic minimal inhibitory concentrations (MICs) were determined by broth microdilution following CLSI guidelines per UCLA’s established protocol M100 35^th^ edition.

### Whole genome sequencing and genomic analysis

Sequencing of hvKp isolates was performed as previously described using the Illumina MiSeq System [7]. More than 0.5 million reads were acquired for each isolate, ensuring the mean depth of whole-genome coverage to be at least 15X (on average > 50X) [13]. Reference strains for the 11 hvKp isolates were determined by KmerFinder (Center for Genomic Epidemiology, https://cge.cbs.dtu.dk/services). CLC Genomics workbench (Qiagen v23.0.4) was used for single nucleotide polymorphism (SNP) analysis, antimicrobial resistance genes via ResFinder Database v1.2 and plasmid presence via PlasmidFinder database. SNP analysis is based on whole genome sequences, including both coding and non-coding regions, as described previously. [7] K-type and multi-locus sequence typing (MLST), were determined using BIGSdb-Pasteur (http://bigsdb.pasteur.fr/klebsiella). ST66 isolates were closely related to a clinical strain (SB5881) from France (NCBI accession: NZ_LR792628.1), ST23 isolates to a clinical strain (TH12908) from China (NCBI accession: CP087123.1), ST3252 and ST2039 isolates to a clinical strain (B055) from Australia (NCBI accession: NZ_CP072200.1). Visualization of isolate alignment with the p-LVPK-like plasmid (NC_005249) was performed using Proksee [14], and the bacterial genome was annotated using Bakta [15]. A Kp isolate was considered hvKp when at least four or more of the following virulence genes are detected: *iucA, iroB, peg-344, rmpA*, and *rmpA2* [4]. For this analysis, the raw sequence reads of each sample were mapped to a concatenated reference genome comprising the five full-length genes using the CLC Genomics Workbench Map Reads to Reference tool with the default setting. A gene is considered detected when the mean depth of gene coverage is >15X and the fraction of reference gene covered is >99% [7].

## Results

### Clinical characteristics

**Fig. 1:**
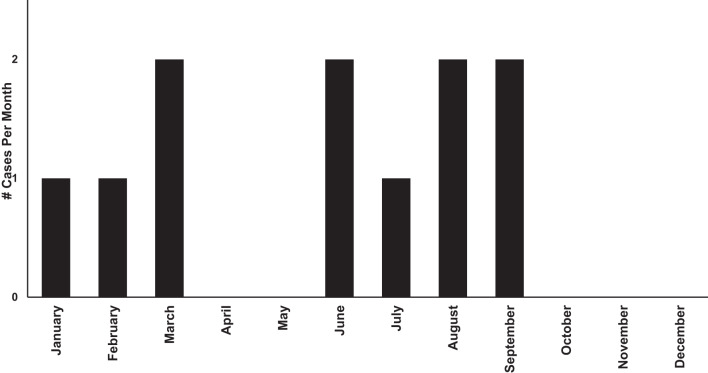
Monthly distribution of hvKp pharyngitis cases. Number of hvKp pharyngitis diagnosed during each month by date of throat culture collection

We identified eleven cases of hvKp pharyngitis between January 2024 and March 2025. Two cases included here were previously published in a case report describing transmission of hvKp within a family unit [12]. Patient demographics are presented in Table S1. Patients were cis-gender female (*n* = 8, 73%) and cis-gender male (*n* = 3, 27%), and ranged in age from thirteen to fifty-eight years old. Four patients (36%) identified as Hispanic or Latino ethnicity, while four (36%) identified as non-Hispanic or Latino. Regarding racial identity, four identified as white/European (36%), one as Filipino (9%), and one as Indonesian (9%). Two patients provided ethnicity but not racial identification, and four provided neither. Most cases occurred during the summer months, between June to September, suggesting seasonal variation in disease incidence (Fig. 1). Of the eleven patients, one resided in Ventura County and the remaining ten resided in Los Angeles County. Within Los Angeles County, patients resided in the Central Los Angeles region (*n* = 6), Westside Cities region (*n* = 3) and San Fernando Valley region (*n* = 2). All patients were diagnosed in an outpatient primary care or urgent care clinic, and none required hospitalization. Patients were generally healthy with few comorbid medical conditions: two patients were pre-diabetic while no patients were diabetic or immunocompromised. Five patients were prescribed chronic intranasal or inhaled corticosteroids. Aside from the cases of family transmission, no explicit sick contacts were documented, although one patient who identified as a man who has sex with men (MSM) described a sexual encounter with a new partner immediately prior to symptom onset. One patient reported recent travel to Hawaii, while recent travel was not reported for the other ten patients.

Clinical characteristics including symptoms, examination findings, and treatment are presented in Table S1. All eleven patients presented with sore throat as the chief complaint. Other commonly reported symptoms were sinus congestion and/or rhinorrhea (*n* = 8, 73%), subjective fever (*n* = 5, 45%), and myalgia (*n* = 5, 45%). Only three patients (27%) reported cough. Gastrointestinal symptoms were uncommon, with diarrhea in one patient and none with nausea or vomiting. Physical examination revealed pharyngeal erythema in about half of patients (*n* = 5, 45%) and pharyngeal exudates in only one patient. Objective fever was only measured in one patient, with a temperature of 38.3 °C during their initial visit. Rapid streptococcal antigen testing was performed in all patients and was negative, prompting throat culture testing by which hvKp was identified. Sputum cultures were not performed in patients with cough. Other diagnostic testing was variable and included influenza testing in seven patients, SARS-CoV-2 testing in six patients, and molecular testing for gonorrhea and chlamydia in two patients. One patient tested positive for Influenza B virus in addition to hvKp, and for the other ten cases testing was negative aside from hvKp isolation.

All eleven patients were prescribed oral antibiotic therapy by their managing physician. One patient was not treated upon initial diagnosis and their symptoms spontaneously resolved, but one month later symptoms recurred, and antimicrobials were prescribed at that time. The most common treatment regimen was amoxicillin/clavulanate 875 mg/125 mg twice daily for ten days (*n* = 8, 73%). In one case, after three days of therapy with amoxicillin/clavulanate, the managing physician recommended changing antibiotic therapy to a fluoroquinolone after the isolate was identified as ‘hypervirulent’ despite broth microdilution showing pan-susceptibility. However, the patient’s symptoms had already improved significantly on amoxicillin/clavulanate and treatment was therefore not adjusted. Separately, the patient with comorbid influenza B infection was initially prescribed amoxicillin/clavulanate, but after five days of treatment presented to a different urgent care with improved sore throat and worsening congestion for which their treatment was switched to levofloxacin. Other treatment regimens included ciprofloxacin for seven days, cephalexin for seven days, and cefdinir for ten days.

Following treatment, none of the eleven patients developed invasive disease or required hospitalization. One patient developed recurrent symptoms one month after treatment with a repeat culture positive for the identical strain of hvKp, which was thought to represent repeat infection from transmission within their family unit as previously described [12]. Repeat throat cultures were collected from an additional three patients within three months after completing therapy, and were negative for hvKp. Therefore, antimicrobial treatment eradicated detectable pharyngeal hvKp in three of four (75%) patients with repeat post-treatment cultures and in all three patients without affected household members.

### Genomic characteristics

**Fig. 2:**
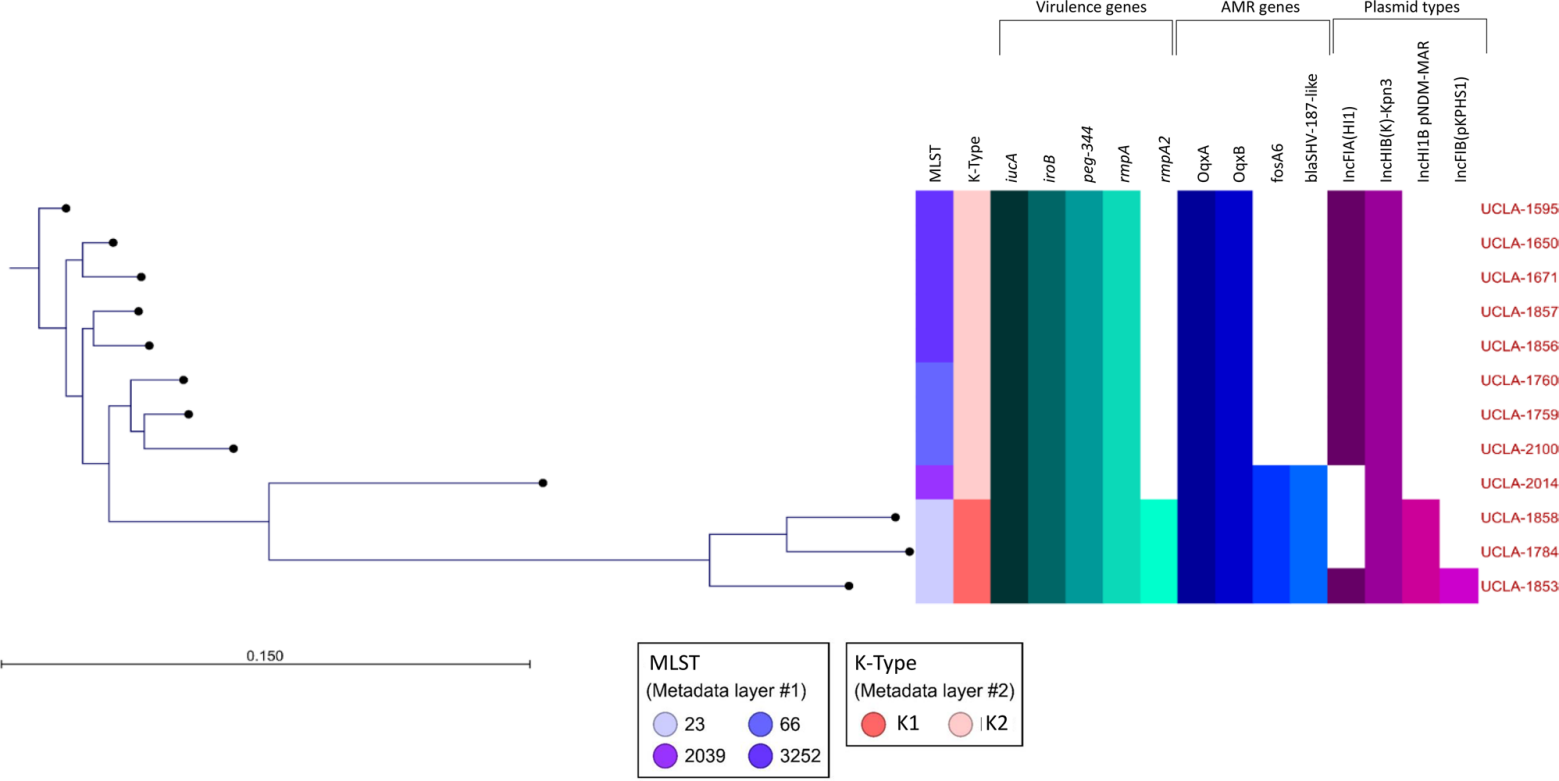
Genotypic analysis of hvKp pharyngitis isolates. Phylogram representing genetic differences amongst isolates (UCLA 1595, 1650, 1671, 1856, 1857, 1760, 1759, 2100, 2014, 1858, 1784, 1853) using K-mer tree clustering based on MLST and K-type, virulence and AMR genes, and plasmid types present in isolates

Twelve hvKp isolates from the eleven cases were characterized by WGS. Two isolates (UCLA-1650 and UCLA-1671) were collected from the same patient at different timepoints. All twelve isolates were string-test positive. Most isolates were K2 serotype (*n* = 9), while the remainder were K1 serotype (*n* = 3). MLST were identified as ST3252 (*n* = 5), ST23 (*n* = 3), ST66 (*n* = 3), and ST2039 (*n* = 1) (Fig. 2).

**Fig. 3:**
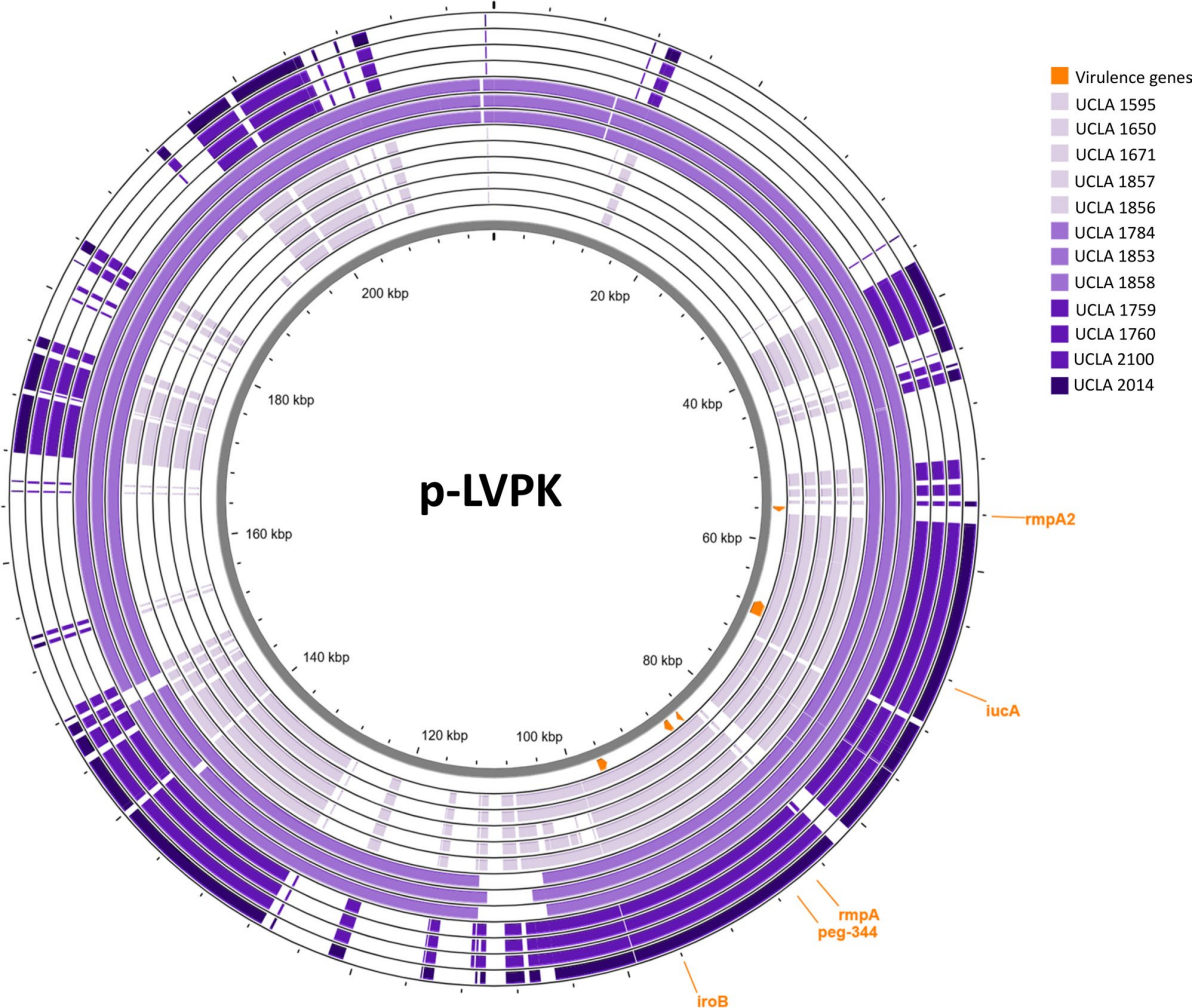
Proksee alignment of all 12 hvKp pharyngitis isolates using the p-LVPK plasmid as reference. The virulence genes category included iroB, peg-344, rmpA, iucA, and rmpA2. Each isolate was categorized into their respective sequence types with group 1 (ST3252) being UCLA 1595, 1650, 1671, 1857, 1856, group 2 (ST23) − 1858, 1784, 1853, group 3 (ST66) − 1760, 1759, 2100, and group 4 (ST2039) − 2014

Sequence identification of virulence genes in Kp isolates accurately identifies strains with in-vivo hypervirulent phenotype [4]. Hypervirulent profiles of our collected isolates were verified by the presence of at least four out of five virulence genes (*iucA, iroB, peg-344, rmpA, rmpA2*). All isolates carried at least *iucA, iroB, peg-344, rmpA*, but only the ST23 harbored *rmpA2* (Figs. 2 and 3). To better visualize hypervirulent gene coverage, we used Proksee to align the twelve isolate sequences to a well-characterized virulence plasmid p-LVPK (Fig. 3). While most isolates displayed a partial alignment to the p-LVPK plasmid, the ST23 isolates showed the highest alignment with coverage of all five virulence genes, including *rmpA2* (Fig. 3).

Furthermore, plasmid analysis revealed all isolates harbored hypervirulence-associated gene-carrying plasmid (*iucA, iroB, rmpA, rmpA2*) IncFIB(K)_Kpn3 [16] with a ≥ 98% pairwise identity, except the ST23 isolates (UCLA-1784, −1853, and −1858) which had a lower pairwise identity of 91.2% (Fig. 2). All ST3252, ST66, and one ST23 isolate (UCLA 1853) harbored the IncFIA(HI1)-type plasmid, carrying *iucA, iroB, rmpA,* and *peg-344*, with a 98.7% pairwise identity (Fig. 2). Additionally, the ST23 isolates harbored an IncHI1B pNDM-MAR-like plasmid, all with a 98.3% pairwise identity, associated with both virulence (*iucA, rmpA/rmpA2*) [17] and Antimicrobial resistant (AMR) genes (*bla*_*KPC-2*_) [16, 17] (Fig. 2). UCLA-1853 was also unique in harboring an additional IncFIB(pKPHS1)-like plasmid, previously linked to a carbapenem-resistance isolate carrying *bla*_*CTX-M-14*_ [18], with a 98.5% pairwise identity (Fig. 2). This specific isolate’s IncFIB(pKPHS1)-like plasmid appeared to be closely related to a well characterized hvKp plasmid pLAKp79, with a 99.8% pairwise identity.

AMR hvKp isolates are an emerging public health threat and can spread within healthcare communities causing difficult to treat invasive infections with high mortality [16, 19]. All hvKp isolates carried *oqxA* and *oqxB* genes, which are associated with fluoroquinolone resistance (Fig. 2). The three ST23 isolates also harbored a beta-lactamase gene *bla*_*SHV-187-like*_ associated with broad-spectrum penicillinase phenotype, as well as the *fosA6* gene associated with fosfomycin resistance (Fig. 2). Interestingly, all isolates tested ‘pan-susceptible,’ showing susceptibility to cefazolin, ceftriaxone, amoxicillin/clavulanate, ciprofloxacin, and trimethoprim/sulfamethoxazole (Table 1). The AMR discrepancy between genotype and phenotype requires further investigation.

**Table 1:** Antibiotic susceptibility results

Antibiotic	MIC	Interpretation†
Piperacillin/Tazobactam	≤8	Susceptible
Cefazolin	1 or 2*	Susceptible
Ceftriaxone	≤1	Susceptible
Amoxicillin/Clavulanate	≤2	Susceptible
Gentamicin	≤1	Susceptible
Ciprofloxacin	≤0.25	Susceptible
Levofloxacin	≤0.5	Susceptible
Trimethoprim/Sulfamethoxazole	≤1/20	Susceptible

* Cefazolin results varied between MIC = 1 and MIC = 2; results were within a normal variation of 2-fold dilutions and both were interpreted as susceptible

†Per CLSI M100 breakpoints

**Fig. 4:**
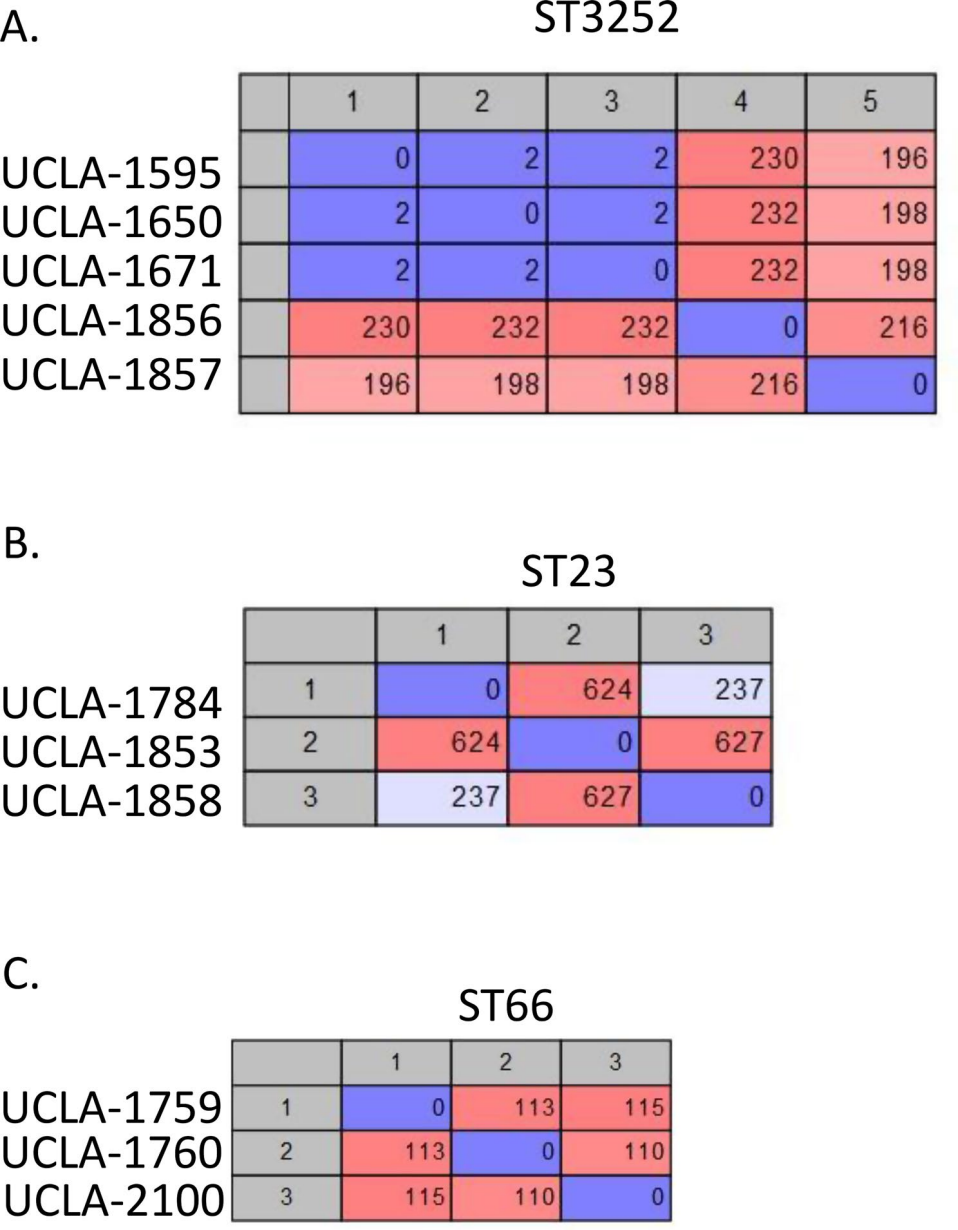
SNP analysis of hvKp pharyngitis isolates, grouped by MLST. **A**. ST3252 isolates SNP comparison - UCLA 1595, 1650, 1671, 1856, 1857. **B**. ST23 isolates SNP comparison - UCLA 1784, 1853, 1858. **C**. ST66 isolates SNP comparison - UCLA 1759, 1760, 2100

SNP analysis was performed on each MLST cluster to assess the genetic relatedness within the ST groups (Fig. 4). Three isolates (UCLA-1595, UCLA-1650, and UCLA-1671) from the ST3252 group were collected from individuals in the same family and possessed < 2 SNP variations indicating high genetic similarity. The remaining two ST3252 isolates (UCLA-1856 and −1857) demonstrated ~200 SNP differences (Fig. 4A). Among the ST23 isolates, UCLA-1853 was highly genetically distinct with >600 SNP differences while the remaining isolates still showed > 200 SNP variation (Fig. 4B). The ST66 group displayed ~100 SNP variation, indicating moderate genetic relatedness, most likely representing a local transmission (Fig. 4C).

## Discussion

HvKp is recognized as a global public health threat for which the WHO recommends increased surveillance [16]. Though hvKp disease was initially associated with endemic spread of the K1–ST23 strain in the Asian Pacific rim, studies in New York, Chicago, and Southern California have identified local acquisition of diverse strains among individuals without recent travel history [6–8]. Similarly, the patients we describe here are of diverse heritage and had not reported recent travel outside of the Unites States. Genes conferring hvKp virulence are borne on mobile genetic elements and are thus transferable between unique strains [1]. As a result, hvKp comprises a serologically and genetically diverse pool of bacteria which are increasingly found to harbor AMR genes [20]. While traditionally understood to cause invasive disease, hvKp also causes non-invasive disease which may be more prevalent than currently appreciated. We propose pharyngitis here, but hvKp may also be implicated in other upper respiratory infections such as sinusitis or otitis media [21–23]. HvKp has been isolated from urine in an asymptomatic outpatient and may be an unrecognized cause of cystitis [24]. Surgical site infections have also been attributed to hvKp, suggesting a possible role for hvKp in non-invasive skin and soft tissue infections [25]. As hvKp becomes increasingly recognized and molecular identification techniques are broadly applied, improved understanding of the full range of hvKp disease is imperative. Of the twelve pharyngeal isolates analyzed in this study, three (1595, 1650, and 1671) were previously described in a brief report that focused on the microbiological characteristics [12]. In this report we re-examined these isolates alongside nine newly identified cases of pharyngitis with isolation of hvKp from throat culture in a >1 year period with expanded genomic, epidemiologic, and clinical analyses.

We propose that hvKp may be the etiologic agent of pharyngitis in these eleven cases but recognize that exhaustive diagnostic testing was not performed. Pharyngitis is most commonly caused by respiratory viruses such as rhinovirus and adenovirus, and the isolation of non-GAS bacteria from throat cultures in symptomatic individuals is often of unclear clinical significance [26, 27]. Still, several bacteria including *Arcanobacterium*, group C *Streptococci*, *Fusobacterium,* and *Klebsiella* species are described to cause pharyngitis [28]. Common symptoms we observed were sore throat, rhinorrhea, sinus congestion, and myalgias. On examination, pharyngeal erythema was present in half of patients while exudates were only seen in one. This presentation overlaps broadly with viral or streptococcal pharyngitis [28]. A weakness of the current study is the lack of uniform testing for common viral pathogens and *Fusobacterium* to more robustly rule out other potential infectious agents. We described one patient who tested positive for influenza B by nasopharyngeal swab PCR in addition to hvKp by throat culture. Pharyngeal hvKp in this patient and others may represent incidental colonization rather than symptomatic infection. Despite this diagnostic uncertainty, the positive culture result prompted antibiotic prescription by all managing physicians.

Antimicrobials have a limited role in the treatment of pharyngitis. Practice guidelines assert that aside from vaccine-preventable or sexually transmitted infections, GAS is the only cause of acute pharyngitis for which antibiotic therapy is explicitly recommended [28, 29]. GAS pharyngitis is highly communicable and can lead to debilitating rheumatic heart disease if untreated. Therefore, treatment with ten days of penicillin therapy is recommended to eradicate GAS from the pharynx [28]. This treatment paradigm for GAS pharyngitis likely influenced the choice of ten days amoxicillin/clavulanate therapy for most of our patients. Antimicrobials are not benign, complications include allergic reactions, gastrointestinal discomfort, and *Clostridioides* difficile infection [30]. Treatment of most non-streptococcal pathogens may also contribute to antimicrobial resistance without providing meaningful benefit [28]. Another pathogen with contested significance is *Fusobacterium necrophorum*: a common cause of pharyngitis in young adults which can lead to Lemierre’s syndrome but is not routinely tested for [31, 32]. The clearest indication for treating pharyngitis is to prevent morbid sequelae.

As discussed above, hvKp is a notable pathogen due to its ability to 1) cause invasive disease and 2) acquire AMR. HvKp stool carriage has been associated with liver abscess caused by colonizing strains [33]. Multiple prospective studies have linked rectal colonization to subsequent invasive infection for cKp [34–36]. Gorrie and colleagues additionally attempted to assess the association between oropharyngeal Kp colonization and subsequent invasive infection, but oropharyngeal colonization rates were exceedingly low [34]. Kp is putatively transmitted by the fecal-oral route. Mouse models suggest that after oral inoculation, Kp replicates in the upper gastrointestinal and respiratory tract before descent to colonize the intestinal lumen [37]. Symptomatic pharyngitis may therefore represent initial oral acquisition of hvKp, which may subsequently colonize the host if untreated. HvKp colonization also has implications for an individual’s close contacts. Case reports have associated asymptomatic carriage of hvKp with development of serious or fatal infections in family members [38–40]. The goal of antimicrobial therapy for hvKp pharyngitis would be not only to alleviate symptoms, but also to prevent invasive disease in both the patient and their close contacts. Our series of eleven patients lacks the sample size, event rate, and untreated controls to address whether treatment impacts the risk for such complications. However, all patients received treatment for hvKp and none developed invasive disease. Ten days of amoxicillin/clavulanate therapy eradicated detectable pharyngeal hvKp in three of four patients with follow-up culture. As the one patient with a positive repeat culture cohabited with affected family members, it is unclear whether the positive repeat culture represents re-infection or persistent colonization. Notably, we did not assess rectal colonization.

Promoting AMR through unnecessary or excessive treatment is also an important consideration. Antibiotics prescribed for respiratory illnesses can lead to enduring colonization with resistant bacterial strains [41]. While our isolates all showed broad susceptibility, outbreaks of carbapenem-resistant hvKp have been reported and carry high mortality rates [19]. Topical antiseptic therapy, such as octenidine mouthwash described by Klaper et al., may present an alternative to systemic antimicrobials [11]. Future studies are needed to assess whether hvKp pharyngitis carries a risk for invasive disease or colonization, and whether this risk is modifiable by treatment.

We attempted to assess risk factors associated with hvKp pharyngitis. Our patients generally had a low burden of medical comorbidities. Diabetes is a known risk factor for invasive hvKp disease, however none of our patients were diabetic and only two (18%) were pre-diabetic [42, 43]. About half (5 of 11, 45%) of patients were prescribed chronic intranasal or inhaled corticosteroids, which has been associated with increased risk for pharyngitis [44]. One patient who identified as MSM described a recent sexual encounter with a new partner prior to symptom onset. His hvKp isolate was K2–ST66. Interestingly, Klaper et al.’s case of hvKp pharyngitis also occurred in a patient described as MSM with recent sexual encounters and the isolate was also identified as K2–ST66 [11]. Surveillance of sexually active adults may clarify whether hvKp is transmitted by sexual activity as these two cases suggest.

WGS of the bacterial isolates revealed a genetically diverse pool of hvKp strains causing pharyngitis, with serotypes and sequence types identified as K2–ST3252 (*n* = 5), K1–ST23 (*n* = 3), K2–ST66 (*n* = 3), and K2–ST2039 (*n* = 1). SNP analysis revealed a > 100 SNP difference between all isolates within each MLST cluster except for three closely related ST3253 isolates collected from patients within the same family unit. Notably, ST2039 isolates have been associated with gut colonization, which can predispose patients for the development and spread of hvKp infection, ultimately occurring within MSM populations [45, 46]. Globally, hvKp infections have predominantly been associated with the K1–ST23 lineage, whereas ST66 and ST3252 are much less frequently reported [1, 47]. In a previous passive surveillance study, we identified K1–ST23 as the predominant strain responsible for invasive hvKp infections in Southern California [7]. Like our current study, these K1–ST23 isolates were highly genetically diverse, indicating multiple circulating strains within the community. One of our K1–ST23 isolates, UCLA-1853, carried multiple plasmids harboring AMR and all five virulence genes which have been implicated in hvKp liver abscesses and meningitis [48]. We also previously identified two K2–ST66 strains in Southern California which diverged by 59 SNPs, indicating a closer genetic relationship than the K1–ST23 isolates. These K2–ST66 strains caused hepatic abscess and bacteremia in one patient, and extensive facial abscess involving parotid gland, middle ear, and endophthalmitis in the other patient [7, 49]. The continuous discovery of K2–ST66 cases in this study further supports this unique hvKp lineage may be circulating in Southern California. The prevalence of K2–ST3252 strains, comprising 40% of hvKp pharyngitis cases, was unexpected. A prior report studying Kp isolate transmission in human and non-human sources from Guadeloupe demonstrated a genotypic analysis where ST3252 belonged to clonal group (CG)-MLST 66 [47]. It is unclear whether the rarity of ST3252 strains in previously published series of invasive hvKp infections reflects a recent emergence of this strain or a predilection of this strain to cause non-invasive disease such as pharyngitis. One possible explanation is the presence of a locally circulating strain, particularly given that patients presenting with pharyngitis may facilitate transmission through close contact. However, other factors, such as sampling bias, limited geographic representation, or temporal clustering, may also contribute to this observation. Moreover, defining hypervirulence based on the presence of ≥4/5 virulence genes required in vivo validation. Importantly, the presence of four rather than five virulence genes was found to have lower predictive value for the hypervirulent phenotype as determined in mouse models [50]. It is possible that the K2–ST66 and K2–ST3252 isolates are less virulent than the K1–ST23 isolates. Due to the limitation of resources, the virulence of these hvKp were not verified using the murine model.

AMR gene analysis in hvKp isolates identified putative fluoroquinolone resistance genes *oqxA* and *oqxB*, in all isolates, with K1–ST23 strains additionally harboring beta lactamase *bla*_*SHV-187-like*_ as well as fosfomycin resistance gene *fosA6.* Historically, ST23 isolates are commonly associated with antimicrobial resistance [51]. These isolates uniquely harbored the IncHI1B pNDM-MAR-like plasmid which carries both hypervirulence and AMR genes, representing the convergence of these two genotypes into a hybrid plasmid [16]. ST3252, along with ST66, share genetically similar plasmids carrying virulence and AMR genes [18, 52–56], distinctive from those genes carried in ST23 [57, 58]. Interestingly, these genetic correlates of resistance were discordant with phenotypic resistance testing by broth microdilution, which demonstrated fluoroquinolone and ceftriaxone susceptibility for all isolates. This discordance may be due to sequence variation within these *bla*_*SHV-like*_ genes which may impact enzymatic activity, particularly with respect to hydrolysis of third-generation cephalosporins. However, as functional validation was not performed, the true phenotypic impact of these variants remains uncertain. These results highlight the importance of phenotypic testing to guide treatment and promote appropriate antibiotic stewardship and further studies to clarify genotype-phenotype correlation of antimicrobial resistance in hvKp.

Our findings in this study are observational and limited by small number of cases (*n* = 11), lack of controls, and lack of exhaustive testing to rule out other causes of pharyngitis. As hvKp becomes increasingly recognized in hospitals worldwide, our findings suggest that larger epidemiologic studies characterizing hvKp isolates obtained from specimens beyond ‘typical’ cases is crucial to understand what factors influence hvKp community spread. Our small case series proposes several hypotheses such as seasonal incidence, association with inhaled or intranasal corticosteroids, and sexual transmission which can be tested in future studies. WGS of hvKp isolates from these eleven pharyngitis cases share similarities in the genetic landscape of isolates causing invasive disease within the community, and reveal new strains otherwise not appreciated by only screening patients with invasive disease. Further studies are needed to verify whether active screening and treatment of symptomatic and asymptomatic hvKp carriage can be of benefit for preventing invasive infections in both patients and their close contacts.

## Electronic supplementary material

Below is the link to the electronic supplementary material.

Supplementary material 1

## Data Availability

The genome sequences generated in this study are available in the GenBank BioProject: PRJNA1179472.

## Abbreviations

AMR: Antimicrobial resistance
CLSI: Clinical Laboratory Standard Institute
cKp: Classical *Klebsiella pneumoniae*
GAS: Group-A *Streptococcus*
hvKp: Hypervirulent *Klebsiella pneumoniae*
MALDI-TOF: Matrix-Assisted Laser Desorption Ionization–Time of Flight
MLST: Multi-locus sequence typing
MSM: Men who have sex with men
NCBI: National Center of Biotechnology Information
SNP: Single nucleotide polymorphism
ST: Sequence type
WGS: Whole-genome sequencing
