# Supplementary material for: Clinical and genomic characteristics of hypervirulent Klebsiella pneumoniae isolated from throat culture suggesting an emerging causative agent of pharyngitis
Source: BMC Infect Dis. 2026 May 26;26:1387. doi: 10.1186/s12879-026-13654-3 (PMC13397599; doi:10.1186/s12879-026-13654-3)
Supplement: Supplementary file 1 — Supplementary material 1 [file 12879_2026_13654_MOESM1_ESM.docx]

**Table S1: Demographic and Clinical Characteristics**

|  | Gender/ Sex | N (%) |
| --- | --- | --- |
|  | Cis-gender female | 8 (73%) |
|  | Cis-gender male | 3 (27%) |
|  | Ethnicity |  |
|  | Hispanic/Latino | 4 (36%) |
|  | Non-Hispanic/Latino | 4 (36%) |
|  | Declined to answer | 3 (27%) |
|  | Race |  |
|  | White/European | 4 (36%) |
|  | Filipino | 1 (9%) |
|  | Indonesian | 1 (9%) |
|  | Declined to answer | 4 (36%) |
|  | County/ Region |  |
|  | Ventura County | 1 (9%) |
|  | LA County, Central LA | 5 (45%) |
|  | LA County, Westside Cities | 3 (27%) |
|  | LA County, San Fernando Valley | 2 (18%) |
|  | Recent travel |  |
|  | Hawaii | 1 (9%) |
|  | No recent travel | 10 (91%) |
|  | Symptoms |  |
|  | Sore Throat | 11 (100%) |
|  | Rhinorrhea/ Congestion | 8 (73%) |
|  | Fever (subjective) | 5 (45%) |
|  | Myalgias | 5 (45%) |
|  | Cough | 3 (27%) |
|  | Diarrhea | 1 (9%) |
|  | Conjunctival Injection | 1 (9%) |
|  | Exam |  |
|  | Pharyngeal Erythema | 5 (45%) |
|  | Pharyngeal Exudate | 1 (9%) |
|  | Fever | 1 (9%) |
|  | Risk Factors |  |
|  | Diabetes | 0 (0%) |
|  | Pre-diabetes | 2 (18%) |
|  | Intranasal or Inhaled Corticosteroid | 5 (45%) |
|  | New sexual partner | 1 (9%) |
|  | Treatment |  |
|  | Amoxicillin/ clavulanate | 8 (73%) |
|  | Cefdinir | 1 (9%) |
|  | Cephalexin | 1(9%) |
|  | Ciprofloxacin | 1 (9%) |
|  | Levofloxacin | 1 (9%) |
|  | Repeat throat culture |  |
|  | Positive | 1 (9%) |
|  | Negative | 3 (27%) |
|  | Not Obtained | 7 (64%) |
